# Case Report: Intestinal *mycobacterium abscessus* infection in a child

**DOI:** 10.3389/fped.2026.1815227

**Published:** 2026-07-20

**Authors:** Linlin Zhang, Dixi Huang, Jie Song, Tianming Zhao, Fan Yang, Chunna Li, Fangfang Zheng

**Affiliations:** 1 Department of Pediatrics, The Fifth Affiliated Hospital of Sun Yat-sen University, Zhuhai, Guangdong, China; 2 Department of Infectious Diseases Service, The Fifth Affiliated Hospital of Sun Yat-sen University, Zhuhai, Guangdong, China

**Keywords:** children, enteritis, *mycobacterium abscessus*, non-tuberculous mycobacterial, NTM

## Abstract

The diagnosis and treatment of *Mycobacterium abscessus* infections present significant challenges, especially in the rare cases of extrapulmonary involvement in pediatric patients. These cases are characterized by diagnostic difficulties, limited therapeutic options, scarce clinical experience, and a lack of evidence-based treatment guidelines. This article reports on a 6-year-old child who experienced fever and abdominal pain. Metagenomic next-generation sequencing (mNGS) facilitated the rapid and accurate identification of *Mycobacterium abscessus* as the causative pathogen. Under a standardized full-course protocol, an individualized therapy regimen (that includes Imipenem, Azithromycin, and Linezolid) led to favorable clinical outcomes. Through the analysis of this successfully treated case, we aim to derive clinical insights and identify potential limitations, with the goal of exploring effective diagnostic and therapeutic approaches for pediatric patients with non-tuberculous mycobacterial (NTM) infections in the future.

## Introduction

Non-tuberculous mycobacteria (NTM) are opportunistic environmental bacteria commonly found in soil and water from both natural and municipal sources. Compared with tuberculosis, NTM has similar clinical symptoms, and there are many pathogenic bacteria, making differential diagnosis difficult and increasing the difficulty of treatment. Globally, NTM incidence is increasing and modeling suggests that, without new interventions, numbers will continue to rise (1). In China, NTM infections in children have been rarely reported, and the incidence rate remains undocumented. Research data from other countries indicate that the prevalence of NTM infections in children ranges from 0.8 to 5.4 per 100,000 (2).

Pediatric NTM disease presents significant diagnostic and therapeutic challenges. Clinically, NTM infections present with nonspecific symptoms, including chronic fatigue, cough, weight loss, and fever. The condition lacks specific clinical manifestations and limited diagnostic techniques contribute to frequent misdiagnosis and widespread diagnostic delays (3, 4). Moreover, the management of the condition is challenged by the scarcity of available treatment options and the requirement for extended durations of therapy. We report a child who was admitted for fever, abdominal pain, and diarrhea. In this child, Metagenomic Next-Generation Sequencing (mNGS) subsequently revealed an infection with *Mycobacterium abscessus*. This case underscores the importance of early confirmation and personalized treatment.

## Case presentation

A 6-year-old boy was admitted to the Fifth Affiliated Hospital of Sun Yat-sen University on April 28, 2024, having experienced a two-week history of fever and diarrhea. His past medical history was unremarkable. Two weeks prior to his admission, he experienced a high fever peaking at 41.0 ℃, which temporarily subsided to approximately 37.5 ℃ after receiving ibuprofen. However, the fever recurred every 5 to 6 h, accompanied by chills and shivering. Additional symptoms included an intermittent dry cough, and sudden episodes of periumbilical abdominal pain lasting several minutes without nausea or vomiting. Following a comprehensive evaluation at a community hospital, he was diagnosed with: Infectious diarrhea with moderate dehydration, Moderate protein-energy malnutrition, Mycoplasma infection and Septicemia and so on. During the third course of azithromycin treatment administered from April 27 to 29, the patient received 0.15 g of azithromycin daily, and the treatment proved ineffective. This ineffectiveness led to his referral to our hospital.

Vital signs on admission were as follows: temperature 39.6 ℃, heart rate 130 beats/min, respiratory rate 22 breaths/min, and blood pressure 110/70 mmHg. The body weight was 16.1 kg, corresponding to a Z-score of −3 to −2 SD (below the 3rd percentile). The patient remained conscious but lethargic, with manifestations of moderate malnutrition and mild dehydration. Ocular examination showed isolated conjunctival injection without eyelid edema, and no superficial lymphadenopathy was identified. The abdomen was scaphoid and soft on palpation, without generalized tenderness or rebound pain. Paroxysmal periumbilical tenderness was induced during abdominal pain episodes, and tympany was noted on abdominal percussion.

**Figure 1:**
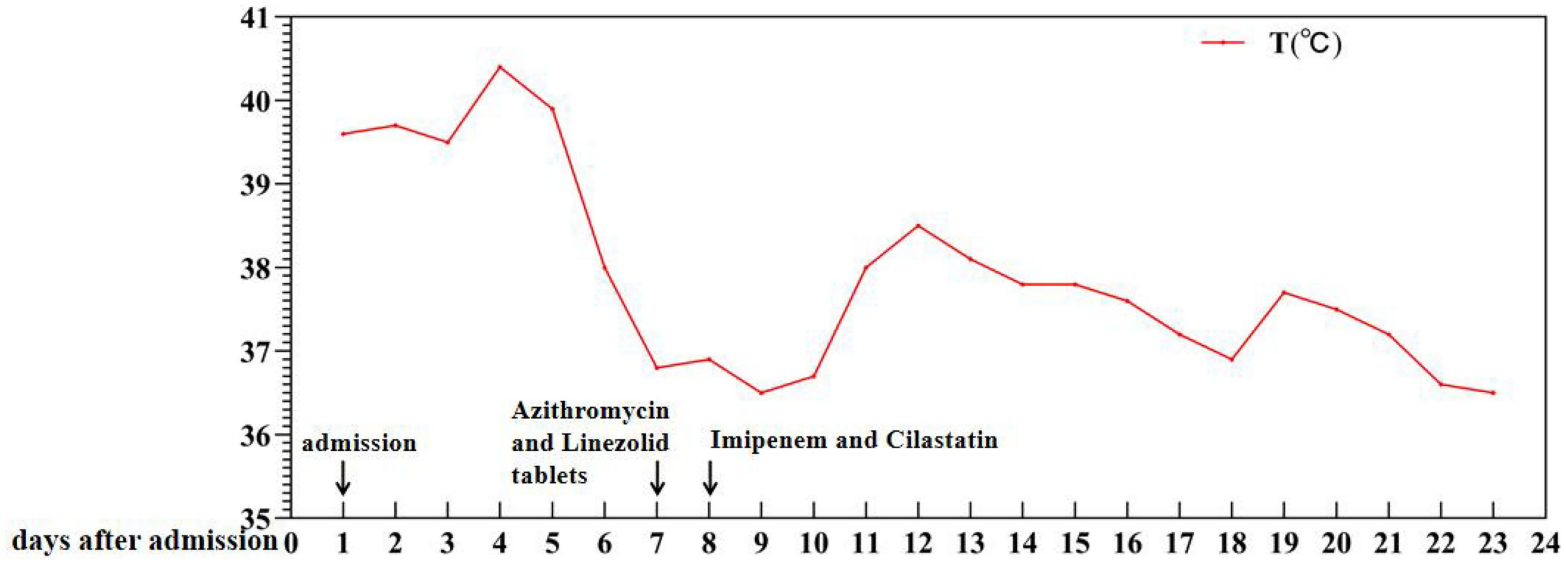
Daily maximum body temperature curve of the patient during the first hospitalization period.

**Figure 2:**
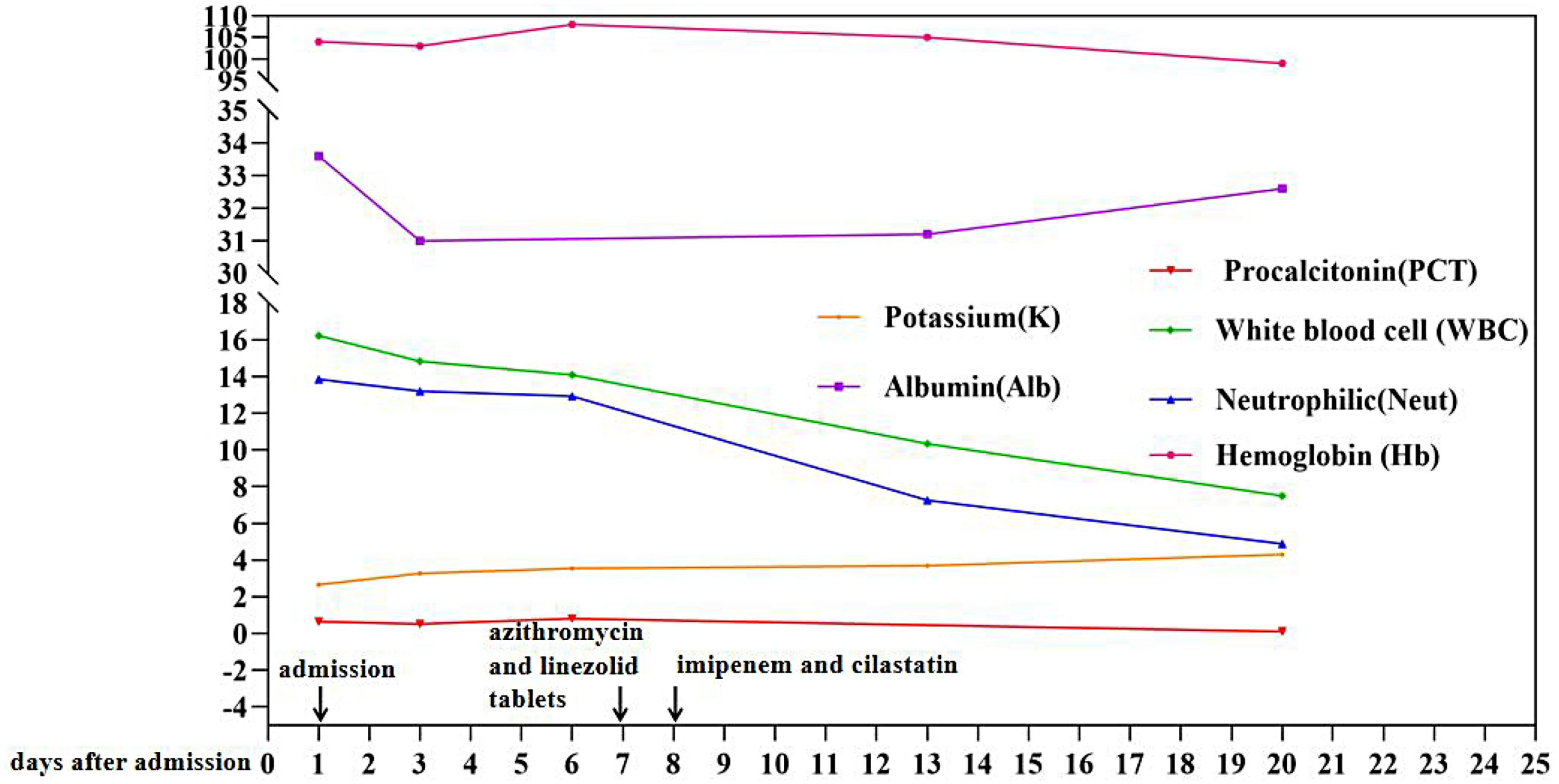
Serial dynamic changes in serum potassium, procalcitonin, serum albumin and routine blood parameters during the patient's first hospitalization.

Initial laboratory examinations on April 28, 2024 (as shown in Table 1) revealed elevated leukocyte counts, anemia, increased inflammatory biomarkers, hypoproteinemia, and abnormal stool results, consistent with severe systemic illness. Chest radiography showed unremarkable findings. Abdominal spiral Computed tomography (CT) demonstrated multiple small intestinal gaseous distension, colonic fecal retention, and mild mesenteric lymphadenopathy. The patient was initially diagnosed with bacterial enteritis. Considering the patient's poor general status and moderate malnutrition, comprehensive risk assessment identified significant procedural risks for colonoscopy, and the examination was therefore temporarily deferred. Empirical antibacterial therapy with piperacillin-tazobactam (1.8 g q8 h) was administered from April 28 to May 3, 2024. Nevertheless, persistent fever, diarrhea, and abdominal pain were observed throughout the treatment course, with no evident clinical improvement. On May 3, the Metagenomic Next-Generation Sequencing (mNGS) of blood showed *Mycobacteroides abscessus* and *Epstein–Barr virus* (*EBV*) infection (as shown in Table 2). The clinical diagnosis was sepsis, a bloodstream infection caused by *Mycobacterium abscessus* and *EBV*. Based on the genetic testing results, the patient was prescribed the following antimicrobial regimen, Linezolid tablets 200 mg every 8 h starting May 4, and Azithromycin 0.15 g once daily starting May 4, Imipenem and Cilastatin 0.24 g every 6 h from May 5 to June 3. After a few days of treatment, the patient experienced significant improvement in abdominal pain and diarrhea, as well as a reduction in body temperature (as shown in Figure 1) and the blood testing (as shown in Figure 2). The laboratory results from May 17, 2024, the 20th day of hospitalization (as shown in Table 1), indicated that infection indicators had decreased from the previous reading, suggesting an improvement in the patient's condition.

**Table 1:** Laboratory results.

Parameter	April 28, 2024	May 17, 2024	Normal range
White blood cell (WBC) (×10^9^/L)	16.24	7.49	4.3–11.3
Neutrophilic (Neut) (×10^9^/L)	13.86	4.88	1.6–7.8
Red blood cell (RBC) (×10^12^/L)	4.31	4.09	4.2–5.7
Hemoglobin (Hb) (g/L)	104	99	118–156
Procalcitonin (PCT) (ng/mL)	0.64	<0.1	0.0–0.5
C-reactive protein (CRP) (mg/L)	51.19	5.06	0–6
Potassium (K) (mmol/L)	2.66	4.31	3.7–5.2
Albumin (Alb) (g/L)	33.60	32.6	39–54
Anti-cyclic citrullinated peptide (anti-CCP) antibodies (U/mL)	<8	/^a^	<17
Ferritin (ng/mL)	223	/	30–400
Rheumatoid factor (RF) (IU/mL)	10.04	/	<14
*Epstein–Barr Virus* (*EBV*) DNA (copies/mL)	Negative	/	<5.0 × 10^2^
*Cytomegalovirus* (*CMV*) DNA (copies/mL)	Negative	/	<5.0 × 10^2^
Occult blood test	Positive	Weak positive	Negative
Fecal leukocytes	++++^b^	Negative	Negative
Vasculitis-related assays	Negative	/	Negative
Blood culture and identification	Negative	/	Negative
Preoperative eight-item panel	Negative	/	Negative
Enteropathogenic bacterial culture	Negative	/	Negative
Urine culture with colony count	Negative	/	Negative
Purified protein derivative (PPD)	Negative	/	Negative

a “/” means not detected.

b “+” means 10 fecal leukocytes per high-power field.

**Table 2:** Pathogenic microorganisms detected using metagenomic next-generation sequencing (mNGS).^a^

Species	Number of detected reads^b^	Confidence level
*Mycobacteroides abscessus*	3,023	99%
*Epstein–Barr virus*	88	99%
*Human herpes virus-6B*	3	/^c^

a mNGS experimental details: Clinical specimen nucleic acid extraction, DNA library preparation and Illumina-based next-generation sequencing were performed following standard laboratory protocols.

b Assay LOD = 5 reads. *Mycobacteroides abscessus* and *Epstein–Barr virus* possess reads well above LOD; *HHV-6B* reads (3) are below LOD. Supplementary genus information from the sequencing report: *Mycobacteroides* (total reads = 3,026, relative abundance = 79.91%), *Lymphocryptovirus* (total reads = 88, relative abundance = 95.74%) and *Roseolovirus* (total reads = 4).

c Not available for confidence owing to reads lower than the detection limit.

**Figure 3:**
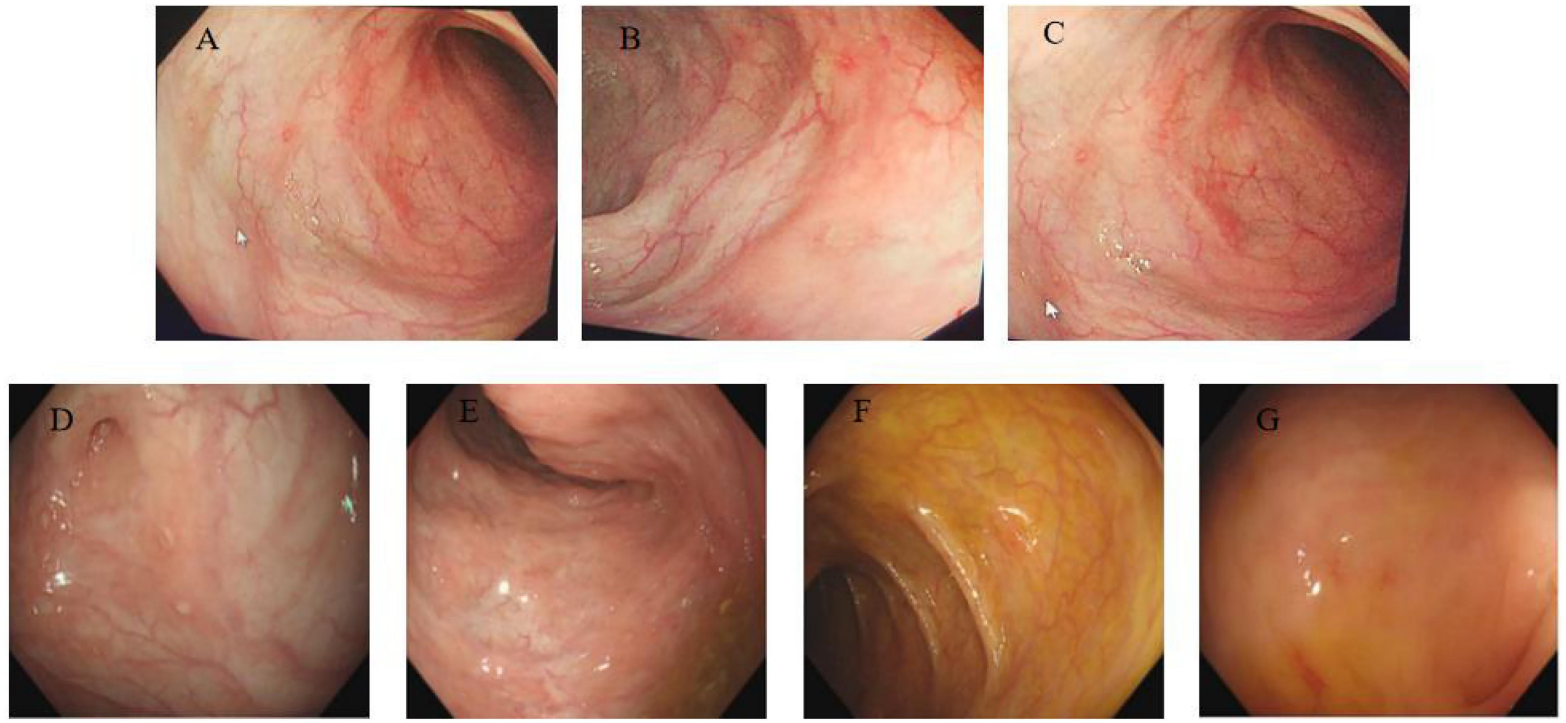
Electron colonoscopy image, dated June 14, 2024, revealed multiple scattered small erosions and ulcerative lesions in the ascending, transverse, descending, and sigmoid colon, measuring approximately 0.2–0.5 cm in size, with a thin white coating **(A)**, dated August 15, 2024, revealed small erosive foci scattered throughout the ascending colon, transverse colon, descending colon, and sigmoid colon, measuring approximately 0.2–0.5 cm **(B,C)**, dated November 18, 2024, revealed focal mucosal erosion of the colonic mucosa, with increased infiltration of lymphocytes, plasma cells, and scattered neutrophils in the stroma, accompanied by localized lymphoid tissue hyperplasia **(D,E)**, dated April 28, 2025, revealing scattered congestion and erosion **(F,G)**.

By June 14, 2024, the patient's clinical condition improved significantly. On initial diagnostic evaluation, colonoscopy (as shown in Figure 3A) identified indeterminate colonic lesions suspicious for ulcerative colitis or intestinal tuberculosis, with a supplementary diagnosis of non-tuberculous mycobacterial (NTM) enteritis.The initial treatment showed favorable efficacy and was therefore continued without modification. The patient was discharged on June 15, 2024, on oral linezolid 200 mg q12 h and azithromycin 0.15 g once daily.

During standardized maintenance treatment, the patient developed recurrent abdominal pain and diarrhea. A second colonoscopy to exclude recurrent lesions performed on August 15, 2024 (as shown in Figures 3B,C) still showed indeterminate colonic lesions. The patient remained afebrile and was otherwise clinically stable.

A third colonoscopy for the follow-up assessment of lesion recovery was performed on November 18, 2024 (as shown in Figures 3D,E). Intestinal tissue culture, mycobacterial identification, and mNGS-based drug susceptibility testing were negative for NTM. Genetic testing for Mendelian susceptibility to mycobacterial disease (MSMD) revealed no pathogenic mutations. Clinical re-evaluation confirmed persistent treatment response and disease improvement. To enhance immune regulation and consolidate therapeutic efficacy, the treatment regimen was adjusted from December 2024 to February 2025. The revised regimen retained linezolid 200 mg q12 h and azithromycin 0.15 g daily, combined with Mycobacterium bovis injection (22.5 *μ*g mycoprotein) administered biweekly for a total of six doses.

**Figure 4:**
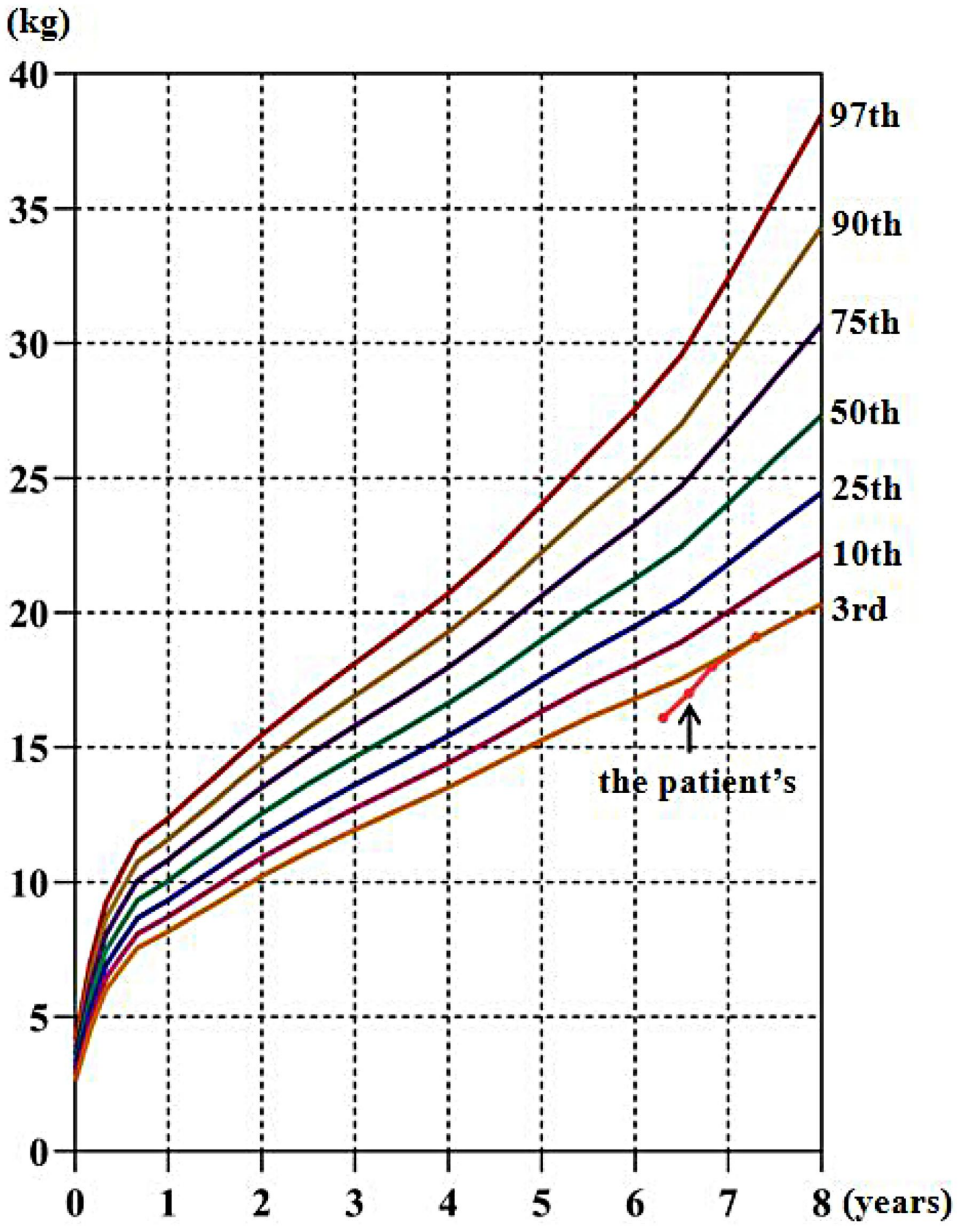
Weight growth percentile curve of the patient plotted against standard weight percentiles for children aged 0 to 8 years.

**Figure 5:**
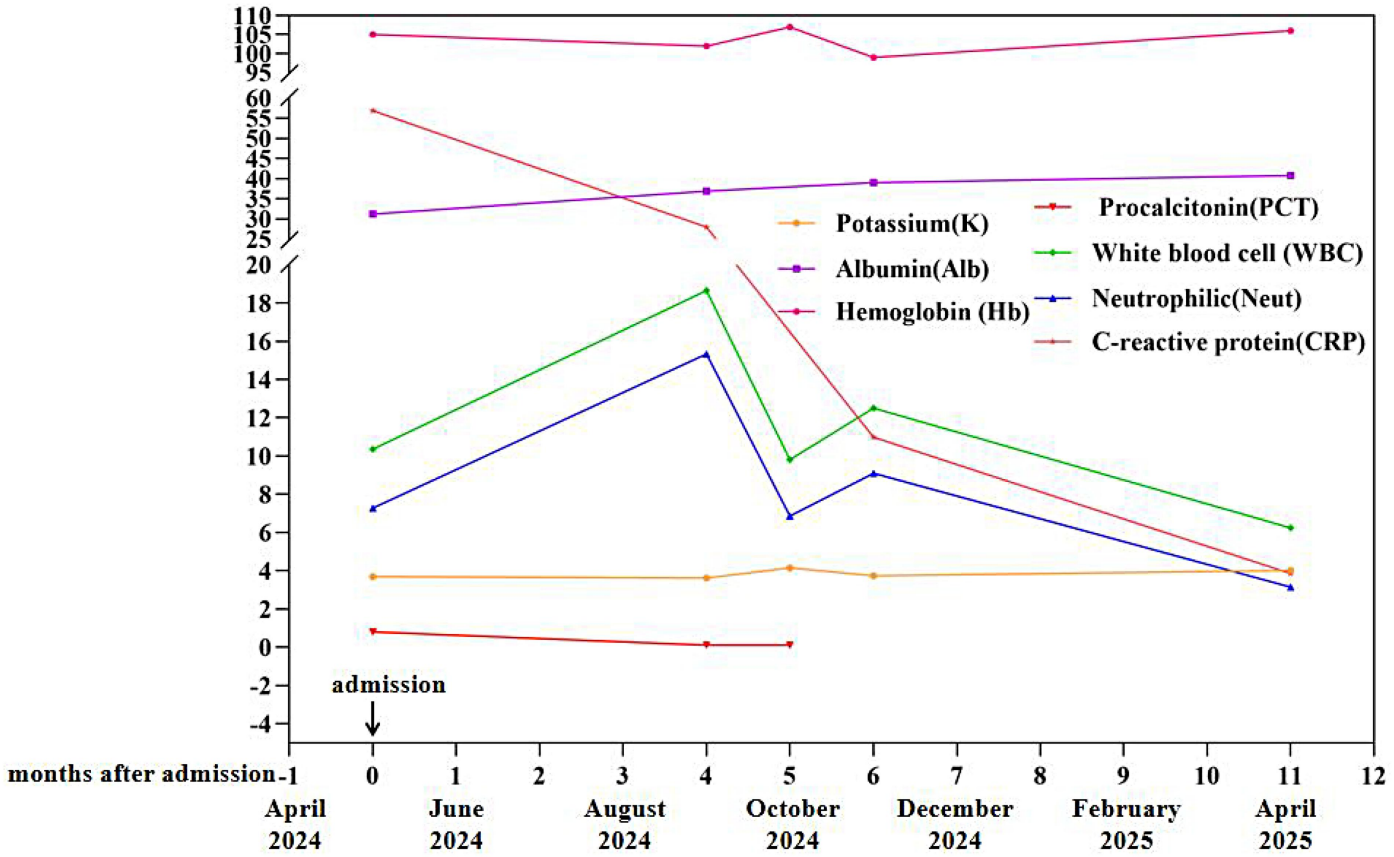
Serial changes in potassium, inflammatory biomarkers, albumin and routine blood parameters during the full treatment period.

A fourth colonoscopy in April 2025 (as shown in Figures 3F,G) demonstrated marked improvement of intestinal lesions. All inflammatory biomarkers returned to normal ranges. No fever, abdominal pain, or diarrhea recurred. The patient showed steady weight gain (as shown in Figure 4) and consistent amelioration of laboratory abnormalities (as shown in Figure 5).

**Figure 6:**
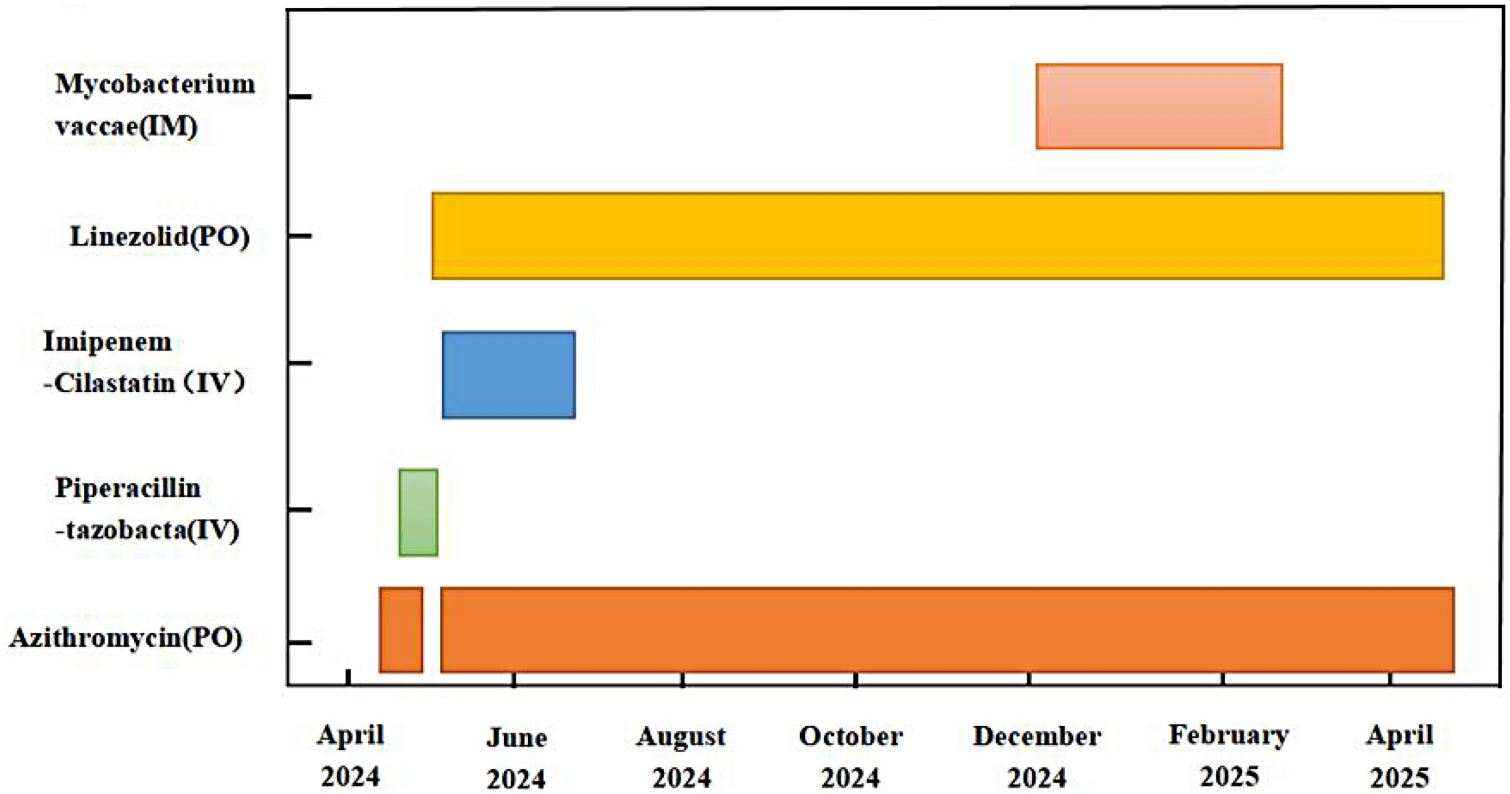
Timeline of antimicrobial treatment administered to the patient throughout the entire clinical course.

All these findings confirmed that the one-year treatment regimen (as shown in Figure 6) was effective with an adequate duration; therefore, medication discontinuation was recommended for close observation and long-term follow-up.

## Discussion

Cervical lymphadenitis represents the most common manifestation of pediatric non-tuberculous mycobacterial (NTM) infection, followed by cutaneous and soft tissue infections. Intestinal NTM involvement is extremely rare in children. Herein, we describe a 6-year-old child who presented with persistent fever, abdominal pain, and diarrhea. Initial laboratory and imaging findings ruled out an acute surgical abdomen. Combined enteroscopy and metagenomic next-generation sequencing (mNGS) successfully identified *Mycobacterium abscessus* (3023 reads), thereby confirming a diagnosis of NTM enteritis complicated with bloodstream infection. No pulmonary lesions were observed on chest computed tomography.

NTM can disseminate to nearly all organs, most frequently the lungs, lymph nodes and skin. Pediatric NTM infections predominantly manifest as extrapulmonary disease (>95% of cases), in stark contrast to adults, who mostly develop pulmonary NTM disease (5). Both pediatric and adult NTM enteritis are scarcely reported. This patient had normal lung structure and no underlying primary or secondary immunodeficiency. Traditionally, NTM infection was thought to occur mainly in patients with structural lung disease or immunocompromise, yet recent studies confirm that immunocompetent individuals are also at risk (6, 7). Early screening for immunodeficiency and timely immune intervention (e.g., *Mycobacterium bovis* vaccine administered in this case) facilitates recovery in NTM patients.

Traditional strain identification methods, such as Roche culture method, were regarded as the “gold standard” in the past, but their time-consuming, complicated operation and high cost limited their wide application in routine laboratories (8). The high-throughput sequencing technique, mNGS has been extensively employed to address the clinical need for rapid diagnostic methods. This approach enables the swift and efficient determination of DNA sequences, offering more precise and comprehensive strain identification outcomes within a reduced timeframe (9). Research conducted by Weiand colleagues (10) on the clinical utility of mNGS in patients suspected of NTM infection demonstrated that mNGS exhibited a sensitivity of 81.4% and a specificity of 97.8%, outperforming conventional culture. High read counts from sterile samples such as blood carry strong diagnostic value, while non-sterile specimens typically require hundreds of reads to confirm pathogenicity (11). In our case, blood mNGS rapidly pinpointed M. abscessus with a high read number of 3023, supporting definitive diagnosis.

Although *Mycobacterium abscessus* accounts for a considerable proportion of NTM isolates in southern China (12), involvement of the intestine is rarely reported. Mycobacterium abscess belongs to fast-growing mycobacteria, which is resistant to most anti-tuberculosis drugs and antibiotics, and it is more difficult to treat than chronic NTM disease (13). Adult regimens rely on 2 or more susceptible antibiotics (amikacin, tigecycline, imipenem, azithromycin, etc.) guided by susceptibility testing, with careful monitoring of drug interactions and comorbidities. No standardized pediatric guidelines exist, and therapeutic options are more restricted: quinolones are contraindicated in children, while combination counts, dosing and adverse reaction tolerance require strict evaluation. Macrolides such as Azithromycin or Clarithromycin and Linezolid are generally considered to be safe for short-term use in children. However, long-term use should be closely monitored for severe, potentially life-threatening toxic reactions, including bone marrow suppression and arrhythmias (14, 15). Imipenem is recommended for the treatment of mycobacterial abscess in the over-the-counter medication guide. Imipenem is the first choice of carbapenem drugs for mycobacterial abscess and mycobacterial turtle, which is generally used for the treatment of NTM disease in the initial stage (intensive phase) for more than one month (16). Tailored to this child's age, infection site and pathogen species, our regimen comprised one month of imipenem-cilastatin plus one-year oral linezolid and azithromycin. Long-term follow-up showed gradual resolution of symptoms, improved weight/nutritional status, normalized inflammatory biomarkers and endoscopic lesions; treatment was successfully completed and discontinued after sufficient therapeutic duration.

Several limitations should be acknowledged. Initial colonoscopy lacked concurrent mNGS, microbial culture, species identification and drug susceptibility testing, which would strengthen diagnostic confidence and guide targeted antibiotic selection. Serial follow-up blood mNGS was also absent, preventing timely confirmation of bloodstream clearance and more precise efficacy assessment. Such comprehensive testing is, however, constrained by patient compliance and economic conditions.

## Conclusions

Non-tuberculous mycobacteria (NTM) has no specific clinical manifestations, and the limited diagnostic methods often lead to frequent misdiagnosis and substantial delays in diagnosis. Overall, the incidence of NTM disease is progressively increasing, and its diagnosis remains challenging due to the complexity of treatment regimens. This is especially true for rare pediatric extrapulmonary NTM infections, where drug options are limited and clinical experience is insufficient. Moving forward, it is imperative to judiciously employ diverse pathogen detection techniques to facilitate rapid and accurate diagnosis of NTM disease, develop tailored treatment strategies, and alleviate patient burden. Ultimately, by analyzing clinical cases with favorable therapeutic outcomes and addressing existing limitations, we aim to advance effective diagnostic and treatment approaches for future pediatric NTM cases.

## Data Availability

The original contributions presented in the study are included in the article/Supplementary Material, further inquiries can be directed to the corresponding authors.
